# Evaluation of the morphometric characteristics of the talus bone-variations and their clinical significance: a descriptive anatomical study

**DOI:** 10.1590/1516-3180.2026.3687.22072026

**Published:** 2026-10-23

**Authors:** Eylem Yalman, Bahadir Murat Demirel, Şükrü Ateş, Emre Şahin, Sema Nihal Küçükoğlu, Seher Yilmaz

**Affiliations:** I Assistant Professor, Department of Anatomy, Faculty of Medicine, Yozgat Bozok University, Yozgat, Türkiye.; II Assistant Professor, Department of Anatomy, Faculty of Medicine, Yozgat Bozok University, Yozgat, Türkiye.; III Assistant Professor, Department of Anatomy, Faculty of Medicine, Yozgat Bozok University, Yozgat, Türkiye.; IV Master’s Student, Department of Anatomy, Faculty of Medicine, Yozgat Bozok University, Yozgat, Türkiye.; V Lecturer, Department of Anatomy, Faculty of Medicine, Erciyes University, Kayseri, Türkiye.; VI Professor, Department of Anatomy, Faculty of Medicine, Yozgat Bozok University, Yozgat, Türkiye.

**Keywords:** Anatomic Variation, Talus, Subtalar Joint, Anthropometry, Talar Facet Classification, Osteometric Analysis, Laterality, Skeletal Asymmetry, Population Variation

## Abstract

**BACKGROUND:**

The talus is the most proximal bone of the ankle and supports the body’s weight. Although its anatomical position and biomechanical role have been widely described, evidence regarding the morphometric characteristics of the talar articular facets is limited.

**OBJECTIVE:**

We aimed to classify the articular facets of the talus and perform morphometric analyses to provide data relevant to orthopedic surgery, rehabilitation, forensic medicine, kinesiology, and anthropometry.

**METHODS:**

In this descriptive anatomical study, a total of 50 dry taluses (29 left, 21 right) of unknown age and gender, acquired from the anatomy laboratories of the Yozgat Bozok and Erciyes universities, were examined. Joint types were classified according to the parameters of the Boyan classification as types A, B, or C, and 16 morphometric measurements were performed. Linear measurements were obtained using a digital caliper, and the angle between the collum tali and corpus tali was measured using a goniometer. Data were analyzed using GraphPad Prism 9; right-left comparisons were made using Student’s t-test, with p < 0.05 considered significant.

**RESULTS:**

Of the taluses, 82% were classified as Type B (B1, 30%; B2, 52%), 14% as Type A (A3, 8%), and 4% as Type C; no Type A4 talus was observed. The right-left differences in anterior-posterior length and transverse width parameters were not significant. However, the lateral articular facet height, caput tali width, talus height, and collum tali angle showed statistically significant differences (p < 0.05).

**CONCLUSION:**

Right-left asymmetries and variations in the type of joint were identified in talus morphometry. Differences in the collum tali angle and joint morphology are important for surgical applications, prosthesis design, and biomechanical analyses. The findings support the anatomical variations described in the literature.

## INTRODUCTION

The foot is the primary support structure of the body and has a complex structure. The bones of the foot are among the most important components of this structure. The foot skeleton comprises 26 bones and contributes to balance, movement, and weight bearing.^1^ The talus is the first bone in the ankle, located most proximally, and supports the weight of the body. The connection it provides between the leg bones and foot bones is of great importance in terms of anatomical position, biomechanical forces, and joint movement.^2^ The talus transmits the weight it supports anteriorly to the metatarsal bones and posteriorly to the calcaneus. For examination, the talus is separated into three parts: head of the talus, neck of the talus and body of the talus; it has no musculotendinous connections.^3^

The articular surface of the head of talus extends forward and inward and articulates with the navicular bone. The neck of the talus is the narrow part between the head and body of talus. The upper surface of the part on the posterior side of the talus, called the body of talus, is pulley-shaped and called the trochlea tali. This surface, which articulates with the facies articularis inferior of the tibia, is called the facies superior.^4^ The medial malleolar articular surface, located on the inner surface of the body of talus, articulates with the tibia, and the lateral malleolar articular surface, located on the outer surface of the body of talus, articulates with the fibula. The projection on the posterior surface of the body is called the posterior process. The posterior process is divided into medial and lateral tubercles by the groove for the flexor hallucis longus tendon. The outer part is called the lateral tubercle, and the inner part the medial tubercle. The neck-body angle, which is located between the long axes of the neck and body of the talus, differs in the talus bones of the right and left feet due to walking habits.^5^ The sulcus tali is located on the inferior surface of the neck of the talus and, by joining with the sulcus calcanei in the calcaneus, forms the sinus tarsi.^6^ The sulcus tali divides this surface into three articular facets: facies articularis calcanea anterior, facies articularis calcanea media, and facies articularis calcanea posterior. These facets articulate with the articular facets located on the upper surface of the calcaneus. The anterior and medial articular facets of the calcaneus, located anterior to the sulcus tali, participate in forming the talocalcaneonavicular joint, whereas the posterior articular facets of the calcaneus, located inferior to the corpus tali behind the sulcus tali, play a role in forming the subtalar joint. Morphological variations in these articular facets can affect subtalar joint movements and impair joint stability, predisposing to ligamentous laxity and arthritis.^2^ ^,^ ^7^ ^,^ ^8^ ^,^ ^9^

The ankle joint is a pulley-like joint located between the lower end of the tibia, the lower end of the fibula, and the talus; it exhibits considerably more complex functions. Because of its unique anatomy, it can support approximately 1.5 times the body weight during walking and 8 times the body weight during running. However, it is the joint that is most frequently affected in sports injuries.^10^ ^,^ ^11^ ^,^ ^12^ ^,^ ^13^ The talocrural joint, formed by the convex articular surface of the talus, along with the subtalar and talocalcaneonavicular joints, forms the ankle joint complex; these joints work together to support movement and the weight of the body.^12^ ^,^ ^13^ The talus articulates with the calcaneus through the articular facets of its inferior surface and distributes the load to the medial and lateral longitudinal arches of the foot via the subtalar joint. Lacking muscular attachment, the talus is the keystone of the passive elements that protect the medial longitudinal arch, and its morphological variations have been associated with flat feet.^10^ The anterior edge of the articular surface of the talus for the tibia is wider than its posterior edge, and the articular surface of the lateral malleolus is wider than that of the medial malleolus. During plantar flexion, the fibula moves distally and shifts anteromedially, rotating inward; with dorsiflexion, the fibula moves proximally and shifts posteromedially, rotating outward. The sharp and wider anterior edge of the talus restricts the ankle during dorsiflexion and forces external rotation. With the help of this structure and ligaments, the talus remains stable during plantar flexion while maintaining mobility. Due to this relationship between the medial and lateral malleoli, the extension of the talus during ankle movements is less than 1 mm.^10^ ^,^ ^11^ ^,^ ^12^ ^,^ ^13^

The biomechanics and stability of the subtalar and talocalcaneonavicular joints depend on the morphological characteristics of the articular facets of the talus and calcaneus. Morphological variants of the articular facets affect the range of motion of the subtalar joint and predispose to the development of ligamentous laxity and subtalar arthritis.^10^ ^-^ ^11^ Bone coalitions, a common cause of the painful condition of flat foot, usually involve the middle articular facet of the calcaneus. The size and shape, morphometric values, and variations in the articular facets of the talus and calcaneus are critically important in the planning of surgical treatments for pathologies such as talocalcaneal and/or subtalar arthritis, talocalcaneal coalition, intra-articular fractures, congenital dysmorphology, flatfoot deformity and valgus deformities. Furthermore, the tarsal bones are the preferred bones for determining gender and age in forensic investigations.^14^ In this study, we aimed to classify the articular facets of the talus; perform morphometric analyses of the talus, which plays a major role in orthopedic surgery, physical therapy and rehabilitation, forensic medicine, kinesiology, and anthropometry; and classify the variations of the articular facets of the talus. Although the talus has been investigated in several morphometric studies, limited data exist regarding articular facet variations and morphometric measurements in the Turkish population.

## OBJECTIVES

This anatomical study was conducted to classify the articular facets of the talus and perform morphometric analyses to provide data relevant to orthopedic surgery, rehabilitation, forensic medicine, kinesiology, and anthropometry.

## METHODS

### Study design and population

This descriptive anatomical study was conducted on a total of 50 (29 left and 21 right) dried talus bones, for which information on age and gender was not specified. The bones were obtained from the collections of the Anatomy Laboratory of Yozgat Bozok University Faculty of Medicine and Anatomy Laboratory of Erciyes University Faculty of Medicine. The sample size was limited to the dry talus specimens available in the anatomy laboratory collections, and the lack of age and gender information should be considered a limitation of the study. This anatomical study was approved by the Yozgat Bozok University Non-Interventional Clinical Research Ethics Committee (meeting date: February 4, 2026; meeting no.: 3; decision no.: 24; decision code: 2026-GOKAEK-263_2026.02.04_24). No pathological findings or traumatic damage were observed at the macroscopic level in any of the examined samples. The talus bones were first classified according to the Boyan classification parameters. Accordingly, each talus joint was classified as one of three types, Type A, B, or C. Type A is divided into four subgroups according to the distance between the facies articularis calcanea anterior and media, and Type B is divided into two subgroups according to the fusion of the facies articularis calcanea anterior and media. The classifications are summarized in Table 1.^15^ Sixteen morphometric parameters of the talus were measured (Figures 1 **and** 2). These measurements included the talus length, talus width, trochlear width, talus height, and collum tali angle, among others. All the parameters measured are presented in Table 2.

**Figure 1.**
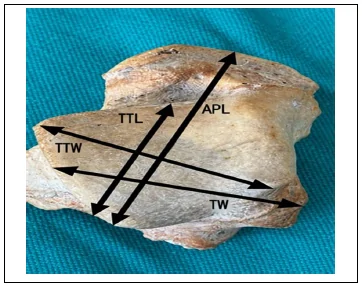
Top view of the talus, showing the anteroposterior length, transverse width, and width and length of the trochlea tali

APL, anteroposterior length; TTL, length of the trochlea tali; TTW, width of the trochlea tali; TW, transverse width.

**Figure 2.**
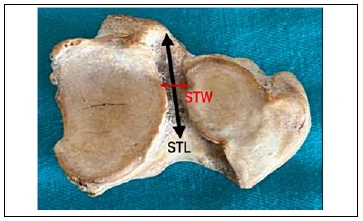
Bottom view of the talus, showing how the width and length of the sulcus tali were measured.

STL, length of the sulcus tali; STW, width of the sulcus tali.

**Table 1.** Talus joint types classified according to Boyan classification parameters

Type	Description
**Type A**	**Anterior, medial, and posterior articular facets of the calcanea are separate**
Type A1	Distance between the anterior and medial articular facets of the calcanea is < 2 mm
Type A2	Distance between the anterior and medial articular facets of the calcanea is between 2 and 5 mm
Type A3	Distance between the anterior and medial articular facets of the calcanea is > 5 mm
Type A4	Only one articular facet is present
**Type B**	**Anterior and medial articular facets of the calcanea are fused, posterior articular facets of the calcanea are separate**
Type B1	Anterior and medial articular facets of the calcanea are partially fused
Type B2	Anterior and medial articular facets of the calcanea are fully fused
**Type C**	**Anterior, medial, and posterior articular facets of the calcanea are fused**

**Table 2.** Parameters of the talus that were measured

Number	Measurement parameter	Abbreviation
1	Anteroposterior length	APL
2	Transverse width	TW
3	Length of the sulcus tali	STL
4	Width of the sulcus tali	STW
5	Depth of the sulcus tali	STD
6	Length of the trochlea tali	TTL
7	Width of the trochlea tali	TTW
8	Width of the medial articular facet	MAFW
9	Height of the medial articular facet	MAFH
10	Width of the lateral articular facet	LAFW
11	Height of the lateral articular facet	LAFH
12	Width of the groove for the flexor hallucis longus tendon	FHLW
13	Depth of the groove for the flexor hallucis longus tendon	FHLD
14	Height of the caput tali	CTH
15	Width of the caput tali	CTW
16	Height of the talus	TH

For linear measurements, all measurements were performed twice by the same investigator, and the mean values of the two measurements were used for analysis. All measuring instruments were calibrated before data collection. Linear measurements, recorded in millimeters, were obtained using a digital caliper with an accuracy of 0.01 mm. Representative linear measurements obtained using the digital caliper are shown in Figures 3 **and** 4. The angle between the collum tali and corpus tali was measured using a digital goniometer. The results are presented as mean ± standard deviation.

**Figure 3.**
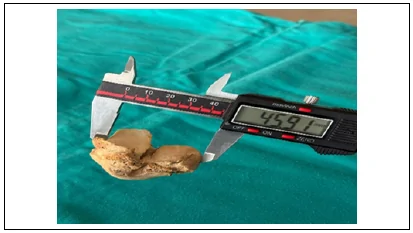
Measuring the anteroposterior length of the talus.

**Figure 4.**
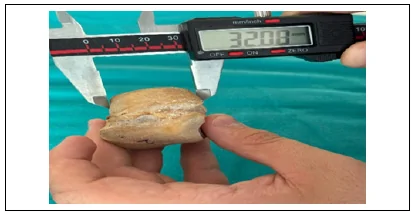
Measuring the width of the trochlea tali.

### Statistical analysis

Normality of the data was assessed using the Shapiro-Wilk test. Right-left comparisons were performed using Student’s t test. Statistical analyses were performed using GraphPad Prism 9. A *p* value < 0.05 was considered statistically significant.

## RESULTS

Seven taluses were classified as Type A (14%; 5 left and 2 right). Type A1 was observed in two specimens (4%; 1 left and 1 right), Type A2 in one specimen (2%; 1 right), and Type A3 in four specimens (8%; 4 left). Type C was observed in two specimens (4%; 1 left and 1 right). Taluses were most frequently classified as Type B (41 specimens, 82%; 23 left and 18 right). Twenty-six were Type B2 (52%; 14 left and 12 right), whereas 15 were Type B1 (30%; 9 left and 6 right). No Type A4 taluses were observed.

The average measurements were as follows: anteroposterior length (left, 53.17 ± 4.19 mm; right, 53.56 ± 3.27 mm), transverse width (left, 39.33 ± 3.26 mm; right, 39.4 ± 2.82 mm), length of the sulcus tali (left, 21.37 ± 3.63 mm; right, 19.98 ± 2.51 mm), width of the sulcus tali (left, 5.82 ± 1.29 mm; right, 6.03 ± 1.2 mm), depth of the sulcus tali (left, 6.3 ± 1.52 mm; right, 6.71 ± 1.19 mm), length of the trochlea tali (left, 30.81 ± 3.05 mm; right, 32.15 ± 2.57 mm), width of the trochlea tali (left, 30.08 ± 2.26 mm; right, 30.33 ± 1.93 mm), width of the medial articular facet (27.64 ± 2.1 mm on the left; 28.51 ± 3.61 mm on the right), height of the medial articular facet (12.59 ± 1.81 mm on the left; 12.21 ± 1.12 mm on the right), width of the lateral articular facet (26.79 ± 2.88 mm on the left; 27.18 ± 2.3 mm on the right), height of the lateral articular facet (left, 22.41 ± 5.24 mm; right, 24.97 ± 2.38 mm), width of the groove for the flexor hallucis longus tendon (left, 8.74 ± 1.4 mm; right, 8.43 ± 1.7 mm), depth of the groove for the flexor hallucis longus tendon (left, 3.49 ± 0.93 mm; right, 3.11 ± 0.91 mm), height of the caput tali (left, 26.44 ± 3.41 mm; right, 26.35 ± 3.39 mm), width of the caput tali (left, 27.34 ± 2.91 mm; right, 29.08 ± 2.21 mm), and height of the talus (left, 30.92 ± 2.92 mm; right, 33.09 ± 2.91 mm). The angle between the collum tali and corpus tali was measured as 53.72 ± 5.48° (left) and 47.52 ± 5.2°(right) (Table 3).

**Table 3.** Results of the morphometric measurements

Measurement parameter	Right	Left	*p*
APL, mm	53.56 ± 3.27	53.17 ± 4.19	0.7214
TW, mm	39.4 ± 2.82	39.33 ± 3.26	0.9324
STL, mm	19.98 ± 2.51	21.37 ± 3.63	0.1396
STW, mm	6.03 ± 1.2	5.82 ± 1.29	0.561
STD, mm	6.71 ± 1.19	6.30 ± 1.52	0.3105
TTL, mm	32.15 ± 2.57	30.81 ± 3.05	0.1094
TTW, mm	30.33 ± 1.93	30.08 ± 2.26	0.6877
MAFW, mm	28.51 ± 3.61	27.64 ± 2.1	0.2842
MAFH, mm	12.21 ± 1.12	12.59 ± 1.81	0.4033
LAFW, mm	27.18 ± 2.3	26.79 ± 2.88	0.6097
LAFH, mm	24.97 ± 2.38	22.41 ± 5.24	0.0426
FHLW, mm	8.43 ± 1.7	8.74 ± 1.4	0.4752
FHLD, mm	3.11 ± 0.91	3.49 ± 0.93	0.1525
CTH, mm	26.35 ± 3.39	26.44 ± 3.41	0.9293
CTW, mm	29.08 ± 2.21	27.34 ± 2.91	0.0267
TH, mm	33.09 ± 2.91	30.92 ± 2.92	0.0127
Angle between collum tali and corpus tali, degrees	47.52 ± 5.2	53.72 ± 5.48	0.0002

APL, anteroposterior length; CTH, height of the caput tali; CTW, width of the caput tali; FHLD, depth of the groove for the flexor hallucis longus tendon; FHLW, width of the groove for the flexor hallucis longus tendon; LAFH, height of the lateral articular facet; LAFW, width of the lateral articular facet; MAFH, height of the medial articular facet; MAFW, width of the medial articular facet; STD, depth of the sulcus tali; STL, length of the sulcus tali; STW, width of the sulcus tali; TH, height of the talus; TTL, length of the trochlea tali; TTW, width of the trochlea tali; TW, transverse width.

Statistically significant differences were observed between the right and left tali for certain parameters. Specifically, significant differences were found in the height of the lateral articular facet, width of the caput tali, height of the talus, and the angle between the collum tali and corpus tali (*p* < 0.05) (Table 3).

## DISCUSSION

In this study, the talus bone was morphologically classified according to joint type and articular facets; various morphometric measurements were also compared between the right and left taluses. Although the data obtained are largely consistent with those reported in the literature, some differences are noteworthy. In the evaluation according to joint type, the rate of Type A accounted for 14% of specimens, with Type A3 being the most common subtype (8%). The low incidences of Type A1 (4%) and Type A2 (2%) suggest that these variants are uncommon in the population. Type A4 was not detected in any sample, which is consistent with its rarity in the literature.^4^ Only two talus samples (4%) were classified as Type C. These data suggest that the distribution of morphological types may differ among populations.^8^ ^,^ ^15^

In the classification according to articular facets, the most common talus type was determined to be Type B (82%); this rate is consistent with the data in the literature.^16^ ^,^ ^17^ The predominance of the Type B2 (52%) indicates that this morphological form is common in the adult population. Type B morphology is reported to be critically important for implant fit in ankle prosthesis and arthrodesis surgeries. In the morphometric analyses, no statistically significant difference was found between the right and left taluses for most parameters. However, significant differences were found in the height of the lateral articular facet, width of the caput tali, height of the talus, and angle between the collum tali and corpus tali measurements. These differences revealed the possibility of morphological asymmetry between the left and right sides, showing that side-dependent measurements should be considered in surgical planning.^18^ In particular, the difference in the angle between the collum tali and corpus tali is clinically important in terms of load distribution and range of motion in ankle biomechanics.^19^ The average values of basic measurements such as the anteroposterior length, transverse width, and length of the trochlea tali obtained in our study are generally similar to those reported in previous morphometric studies.^2^ ^,^ ^20^ ^,^ ^21^ However, the differences observed in some parameters, such as the length of the sulcus tali and height of the lateral articular facet, may be related to factors such as ethnicity, age, gender distribution, and sample size.^22^ ^,^ ^23^

The data we collected revealed that significant lateral asymmetries are present in talus morphometry and that the distribution of joint type may vary depending on the population. Taking these findings into account in clinical applications can improve the success of surgical and prosthetic designs. A good understanding of the specific anatomical structure and morphometric values of the talus and its joint type, and the unique ­biomechanical properties arising from these characteristics, will increase the success rate of surgical and anesthetic interventions in this region and contribute to minimizing possible complications. In this context, by revealing morphological differences between the right and left talus, our findings may provide a useful basis for future studies.

## CONCLUSION

This morphometric evaluation of the talus demonstrated that the predominant joint morphology was Type B, whereas types A and C were less frequently observed. Significant right-left asymmetries were identified in the lateral articular facet height, caput tali width, talus height, and angle between the collum tali and corpus tali. These anatomical variations are critically important for orthopedic surgery, prosthesis design, subtalar and talocrural joint biomechanics, and rehabilitation planning. Awareness of population-specific morphometric characteristics and side-dependent differences can enhance surgical precision, improve implant compatibility, optimize biomechanical assessments, and reduce postoperative complications. These ultimately contribute to better clinical outcomes.

## Data Availability

The datasets generated and analyzed during the current study are available from the corresponding author, Eylem Yalman, upon reasonable request.
